# Artificial Respiration—Fell’s Method

**Published:** 1888-03

**Authors:** 


### *ARTIFICIAL RESPIRATION—FELL'S METHOD.*

In this number of the JOURNAL, Dr. Fell reports the second case of narcotic poisoning treated by forced respiration. Subsequent to that, he has had a third and most interesting case, the details of which have been reported in the daily papers. This last operation was one in which publicity could not be avoided, and we were pleased to note the handsome treatment Dr. Fell received from the secular press. With effusive praise and excusable ignorance, however, some of the editors would rank Dr. Fell with Lister, McDowell and Pasteur, and the medical press has been criticized that it has not joined in the glorification of the Doctor.

To the medical man, however, artificial respiration, employed in cases of poisoning, drowning, etc., is no new thing. The opening of the trachea, and compelling respiration by means of the bellows, is a familiar operation to most medical students. The application to man, of this forced respiration, in those cases in which the nervous energy of the respiratory center is approaching exhaustion, is, we believe, original with Dr. Fell.

If experience proves that this expedient is really a valuable one, and an addition to the means of saving life which the profession has not hitherto utilized, then we will most heartily congratulate the Doctor on having achieved a place with those who have added something to the common store of medical knowledge. Few, indeed, in the present advanced stage of medical science, are so fortunate as to gain this great distinction.

The actual value of new methods of treatment can never be at once appraised, and the profession is notably conservative in receiving such additions; at first, they are apt to be magnified by the halo of novelty and the enthusiasm of the ignorant; possibly, a real discovery may, for a time, seem to be ignored, but the reward never fails to come to him who is worthy of it.

It is with extreme regret that we learn that Dr. Fell has patented the instruments and appliances used by him in his operations. By this act, he has allied himself with those in the profession, of mercenary instincts, who, having happened upon some new means of benefiting humankind, would sell that knowledge to the highest bidder, or would conceal it from others, unless for a pecuniary consideration. In doing this, they violate a law stronger than any printed code, a law which we hold by inheritance, and which has ever been upheld by the faithful physician. It carries with it a sense of common rights, of common knowledge, of joint relationship, of unselfish devotion to the welfare of the profession and humanity, of faithful performance of duty, regardless of pecuniary reward.

The laity may sneer at our ethics and deride our unbusiness-like methods, but this unwritten code stands as a mute guardian to the community. Because of it, our profession recoils from mean ways, loathes trickery, and shrinks from charlatanism in all its forms. A physician may ignore it, he may worship Mammon, he may accumulate riches—he may even become a congressman—but can all this compensate him for the feeling that he is despised of his brethren; " tumbled down from the top of honor to disgrace's feet "?

